# Supplementary material for: Sequence-to-sequence models with attention mechanistically map to the architecture of human memory search
Source: Commun Psychol. 2025 Oct 14;3:146. doi: 10.1038/s44271-025-00322-6 (PMC12521410; doi:10.1038/s44271-025-00322-6)
Supplement: Supplementary file 2 — Supplemental Information [file 44271_2025_322_MOESM2_ESM.pdf]

Supplementary Information

S1 Supplementary Methods

S1.1 Additional Model Details

For all seq2seq models in both the individual fitting and optimization experiments, the encoder and decoder GRUs each use a hidden dimension size of 32, 64, or 128, depending on the experiment, and the word embeddings are 50-dimensional pre-trained GloVe vectors. The total number of trainable parameters in the seq2seq model across experiments is 99,184, 162,704, and 332,752 for the 32, 64, and 128 hidden dimension-sized models, respectively.

To capture human recall data with this seq2seq model, we set up an output/retrieval rule that aligns closely with that of CMR. Similarly to how M^{CF} stores the associations between contexts and their corresponding items (see Eqn. 12 in the main text), the seq2seq model stores an episodic memory table, with keys being the encoding contexts (h_i) and values being the items' semantic embeddings (x_i) obtained through application of GloVe embeddings to the original items f_i. This table serves as a context-dependent memory store, emulating the retrieval rule found in CMR, and ensuring that the model's recalls are constrained to only items that appeared in the presented list. At each decoding step j, the decoder receives the semantic embedding of the previously recalled item (x_j) as input and updates its internal hidden state (see Eqn. 2 in the main text). After the attention mechanism is applied (as in Eqn. 5 in the main text), the final hidden state $\hat{h}_j$ is compared to each encoder hidden state h_i stored in the memory table using cosine similarity: $\text{sim}(\hat{h}_j, h_i) = \frac{\hat{h}_j \cdot h_i}{|\hat{h}_j||h_i|}$. The retrieval process then computes a weighted combination of all stored embeddings x_i, where the weight of each embedding is proportional to its similarity with the current context $\hat{h}_j$: $r_j = \sum_{i=1}^{L} \text{sim}(\hat{h}_j, h_i) \cdot x_i$, where r_j represents the word embedding retrieved from the episodic memory table. To convert r_j into a discrete item, it is passed through a pre-trained inverse embedding, implemented as a two-layer MLP with ReLU activation: $\hat{f}_j = W_2 \,\text{ReLU}(W_1 r_j)$, where $W_1 \in \mathbb{R}^{512\times d}$, $W_2 \in \mathbb{R}^{|\mathscr{V}|\times 512}$, d is the embedding dimension, and $|\mathscr{V}|$ is the vocabulary size. The MLP is trained for 200 epochs using cross-entropy loss to reconstruct the original one-hot encoded word from its GloVe embedding. This one-hot vector f_j represents the final prediction for the current decoding step and serves as the input for the next decoding step.

S1.2 Additional Model Training Details

When obtaining optimal model behavior, prior to reinforcement learning, we pre-trained the seq2seq models in a supervised fashion using randomly generated lists of words appearing in the PEERS dataset vocabulary. This supervised pre-training phase initializes the seq2seq models with useful representations and stable behaviors before reinforcement learning, improving sample efficiency and mitigating exposure bias—where models perform significantly better on input items that appeared more frequently in training—which has been shown effective in sequence prediction and neural machine translation tasks[1,2]. The model was trained to output the entire recall set for each input list, irrespective of item order. To accomplish this, we used a set prediction loss based on the differentiable Sinkhorn algorithm[3,4]. For each training batch, we first computed the cross-entropy loss between each predicted token and every target token, resulting in a cost matrix C. The Sinkhorn-Knopp algorithm was then applied to $-C/\tau$, where the temperature parameter τ (set to 1.0 in our experiment) controls the sharpness of the soft assignment between predictions and targets. After 20 Sinkhorn iterations, this yields a doubly-stochastic matching matrix P, and the final set loss is defined as: $L_{\text{set}} = \frac{1}{B}\sum_{b=1}^{B}\sum_{i=1}^{N}\sum_{j=1}^{N} P_{ij}^{(b)} C_{ij}^{(b)}$, where B is the batch size and N is the sequence length. We used a batch size of 32, Adam optimizer with a learning rate of 0.001, and trained for a total of 10 epochs. No dropout or gradient clipping was used in training across any of the experiments. For comparison of optimal model behavior and human data, we use the vocabulary from experiments in the PEERS free recall dataset[5]. For pre-training, all model configurations were trained using 50,000 randomly generated sequences of 14 words (no repeats) sampled from the PEERS vocabulary. The weights learned during this pre-training phase were used to initialize the model for subsequent reinforcement learning.

S2 Supplementary Discussion

S2.1 Examining Alternative Model Architectures without Attention

To evaluate whether the benefits of the attention module arise from its underlying mechanism or merely from its increased parameterization or model complexity, we trained and evaluated a seq2seq model in which the standard attention layer was replaced by a dense layer operating only on the decoder hidden states (with no access to encoder states). We then repeated the same analysis reported in the main results in Figure 6. As shown in Figure S1 A–C, the dense-layer model produced serial position, probability of first recall, and conditional response probability that were qualitatively similar to those of the

model without attention (i.e., with the standard attention layer removed). Both models exhibited deficits in recall performance (Figure S1 A) and showed no backward contiguity effects in any stage of training (Figure S1 C) as compared to the model with the attention mechanism. These results confirm that the advantage of attention in our framework is its ability to access prior encoding contexts, not simply due to increased parameterization or model complexity.

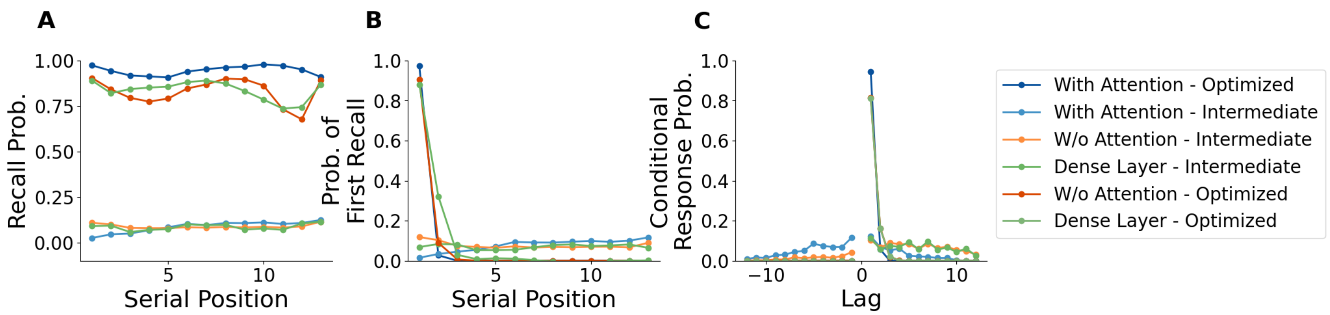

Figure S1. Behavioral patterns of the seq2seq model with attention, without attention, and with the attention module replaced by a dense layer operating only on the decoder hidden states, optimized or during intermediate training. Models are compared across three sets of patterns: serial position curve (A), probability of first recall (B), and conditional response probability (C). The seq2seq model with a dense layer shows similar performance and behavior to the model without the attention mechanism. Both models show reduced recall performance (A) and a lack of backward contiguity as compared to the model with attention (C). Model hidden dimension size is 64. (N=20 for all analyses.)

S2.2 Cross-validation Study of Individual Participant Fitting

To assess whether different methods of conducting the training and testing splits would affect our conclusion that seq2seq performs better than CMR in predicting recall behavior, we conducted a 5-fold cross-validation on a random subset of 20 participants from the full dataset of 171 participants given our time and compute constraints (A full 5-fold cross-validation run for a single participant requires on average 17.5 GPU-hours). In this cross-validation, the dataset is randomly divided into five equal parts, and the model is trained on four parts while the remaining part is used for validation and testing; this process is repeated five times so that each part serves as the validation and test set once. To maintain consistency with our original 90%-5%-5% train-validation-test splits, we used the standard 5-fold cross-validation with an 80%-20% train-test split. After designating 80% of the data for training in each fold, we randomly sampled two non-overlapping 5% subsets – one for validation and one for testing – from the remaining 20% of data. The seq2seq model with attention was trained and evaluated using the same procedures as in the main analysis. Model performance was assessed on held-out folds using root-mean-square error (RMSE) across the three behavioral measures (the serial position curve, the probability of first recall, and the conditional response probability) as in our original analysis depicted in Figure 3. Figure S2 shows the RMSE for each cross-validation fold relative to the corresponding participant's recall patterns (averaged across all behavioral measures), with the RMSE for CMR included for comparison.

We see that a significant difference between seq2seq and CMR RMSE remains after the cross-validation study, corroborating the results of our main analysis. To ensure this difference was significant, we conducted a Wilcoxon signed-rank test comparing the RMSEs averaged across all seq2seq model splits with the CMR RMSEs (two-sided: $W = 0$, $n = 20$, $p = 1.907 \times 10^{-6}$, ranked biserial correlation = -0.989). This result indicates that the seq2seq models exhibit significantly lower RMSE values on average compared to CMR. These findings support that different methods of conducting the training and testing splits do not affect our conclusions in model comparison.

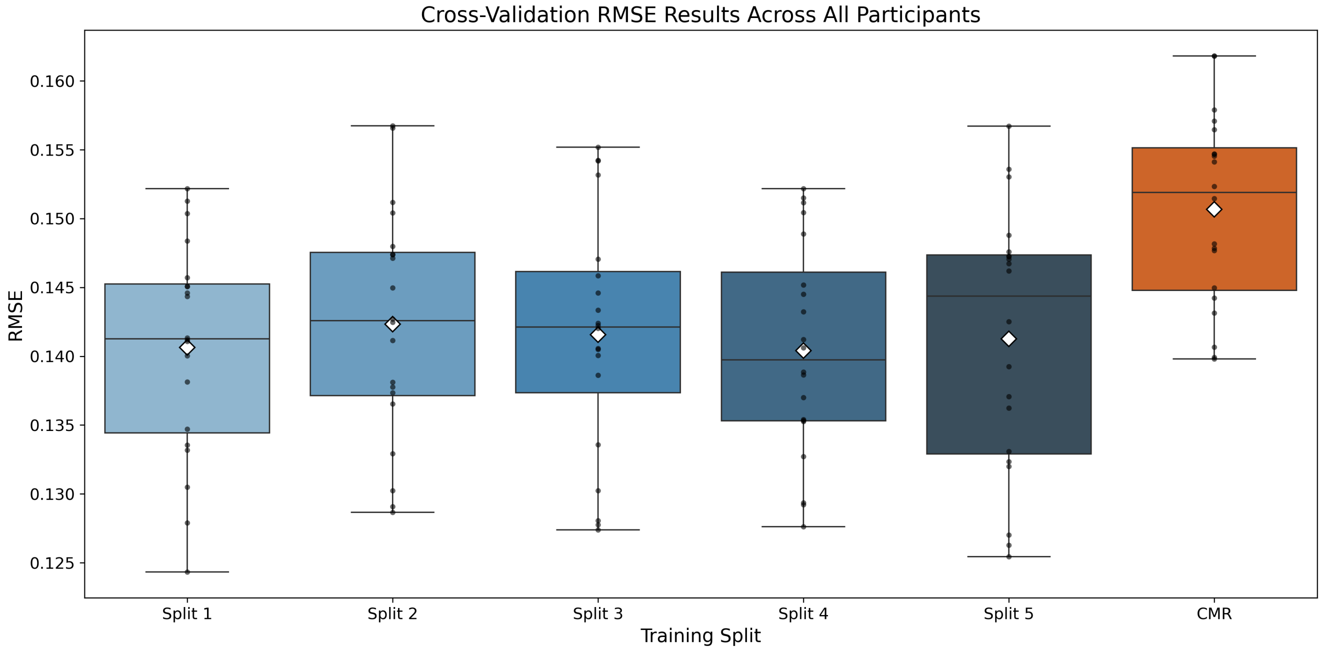

Figure S2. 5-Fold cross-validation results of individual participant fitting for the seq2seq model with attention and CMR. Model performance was assessed on held-out folds using root-mean-square error (RMSE) across all three behavioral measures (the serial position curve, the probability of first recall, and the conditional response probability). (N=20 for all analyses.)

S2.3 Individual Participant Fitting Under Different Training Dataset Sizes

To assess how the amount of training data influences the ability of the seq2seq model (with attention) to outperform CMR in fitting individual-level recall data, we compared the fit quality of both models across different training data sizes. We trained and evaluated models on 20 randomly selected participants using three different train-validation-test splits: 90-5-5, 60-20-20, and 40-30-30. For each split, models were fit to individual participants and assessed on root-mean-square error (RMSE) across the three sets of recall behavior metrics (the serial position curve, the probability of first recall, and the conditional response probability). While the seq2seq model utilizes all three train-validation-test splits, the CMR model utilizes only the training set and the testing set, as a validation set is not required during the CMR model fitting. Both the seq2seq model and the CMR model have equal amounts of training and testing data for comparison. Figure S3A displays the average RMSE across all tasks for each data split across all 20 participants included in the study.

Results show that whether the seq2seq model outperforms CMR depends on the training data size. Pictured in Figure S3B-D are the distributions of RMSE values for the serial position curve, the probability of first recall, and the conditional response probability, respectively, across all splits. In the 90-5-5 regime, the seq2seq model significantly outperformed CMR, yielding lower RMSE across participants (Wilcoxon signed-rank test: two-sided, $W = 8.0$, $n = 20$, $p = 4.77 \times 10^{-5}$, ranked biserial correlation = -0.924). However, as the amount of training data decreased, this advantage was reversed. In both the 60-20-20 and 40-30-30 splits, CMR significantly outperformed the seq2seq model (60-20-20: two-sided, $W = 0.0$, $n = 20$, $p = 1.91 \times 10^{-6}$, ranked biserial correlation = 0.995; 40-30-30: two-sided, $W = 5.0$, $n = 20$, $p = 1.91 \times 10^{-5}$, ranked biserial correlation = 0.952). This crossover indicates that CMR achieves better fits than seq2seq when the available training data is limited. These results support the conclusion that neural network models, such as our seq2seq model, can surpass traditional models given ample training data, but established cognitive models like CMR are more efficient in scenarios where training data is limited.

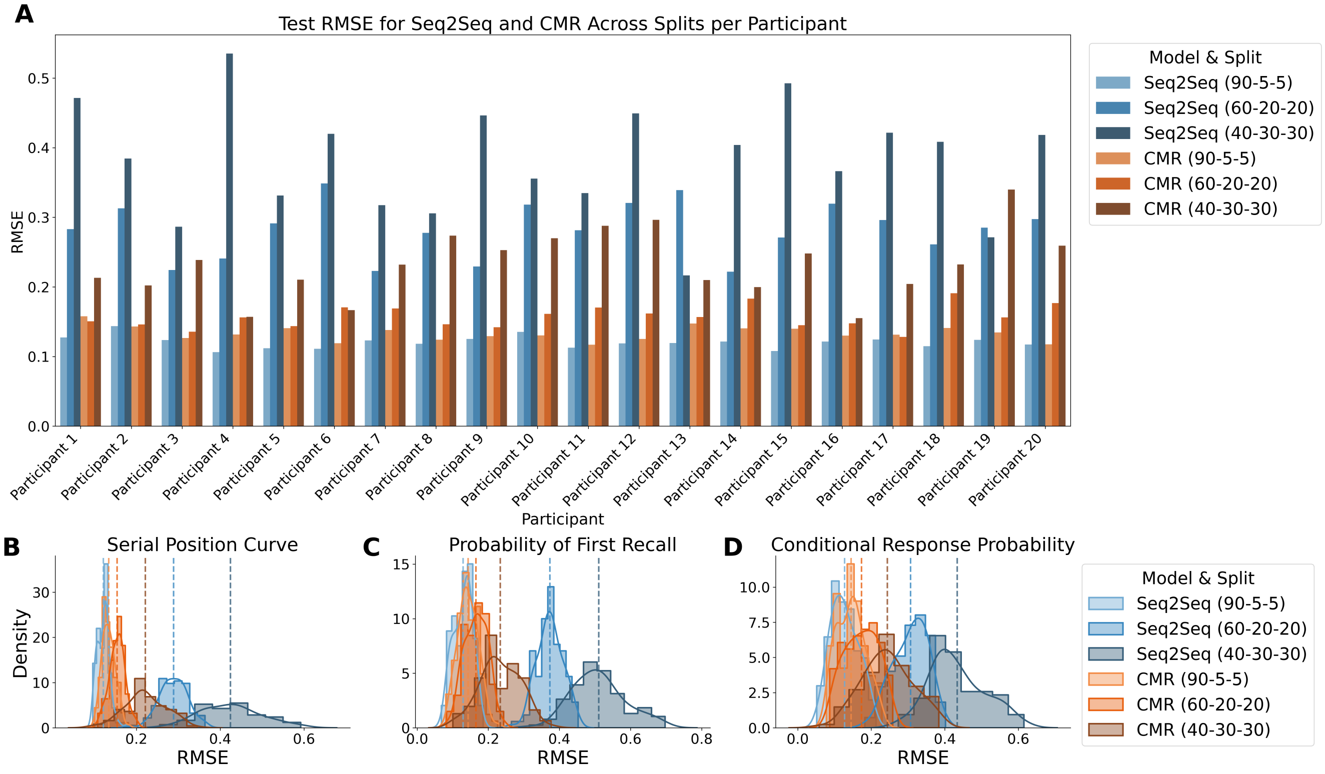

Figure S3. Individual participant fitting results for the seq2seq model with attention and CMR across different amounts of training data. (A) Each grouped set of bars represents the root-mean-square error (RMSE) between the seq2seq model with attention or the CMR model and the human participant data for a given data split for each participant. The seq2seq model with attention exhibits lower RMSE values than CMR with ample training data (90-5-5 split) but higher RMSE values with less training data (60-20-20 and 40-30-30 splits). (B-D) Distributions of RMSE values for the serial position curve, the probability of first recall, and the conditional response probability, respectively, across all splits.

Supplementary References

1. Bahdanau, D. *et al.* An actor-critic algorithm for sequence prediction. *ArXiv* **abs/1607.07086** (2016).
2. Ranzato, M., Chopra, S., Auli, M. & Zaremba, W. Sequence level training with recurrent neural networks. In *4th International Conference on Learning Representations, ICLR 2016* (2016).
3. Cuturi, M. Sinkhorn distances: Lightspeed computation of optimal transport. In *Neural Information Processing Systems* (2013).
4. Mena, G., Belanger, D., Linderman, S. & Snoek, J. Learning latent permutations with gumbel-sinkhorn networks. In *International Conference on Learning Representations* (2018).
5. Kahana, M. J. *et al.* The Penn electrophysiology of encoding and retrieval study. *J. Exp. Psychol. Learn. Mem. Cogn.* (2022).
